# Uncovering the genetic basis for early isogamete differentiation: a case study of *Ectocarpus siliculosus*

**DOI:** 10.1186/1471-2164-14-909

**Published:** 2013-12-21

**Authors:** Agnieszka P Lipinska, Sofie D’hondt, Els JM Van Damme, Olivier De Clerck

**Affiliations:** 1 Phycology Research Group and Center for Molecular Phylogenetics and Evolution, Ghent University, Krijgslaan 281, Building S8, 9000 Ghent, Belgium; 2 Department of Molecular Biotechnology, Laboratory of Biochemistry and Glycobiology, Ghent University, Coupure Links 653, 9000 Gent, Belgium

**Keywords:** Gamete, Transcriptome, *Ectocarpus*, Sexual reproduction, Isogamy, Brown alga, Signaling

## Abstract

**Background:**

The phenomenon of sexual reproduction characterizes nearly all eukaryotes, with anisogamy being the most prevalent form of gamete discrimination. Since dimorphic gametes most likely descend from equal-sized specialized germ cells, identifying the genetic bases of the early functional diversification in isogametes can provide better understanding of the evolution of sexual dimorphism. However, despite the potential importance to the evolutionary biology field, no comprehensive survey of the transcriptome profiling in isomorphic gametes has been reported hitherto.

**Results:**

Gamete differentiation on the genomic level was investigated using *Ectocarpus siliculosus,* a model organism for brown algal lineage which displays an isogamous sexual reproduction cycle. Transcriptome libraries of male and female gametes were generated using Next Generation Sequencing technology (SOLiD) and analyzed to identify differentially regulated genes and pathways with potential roles in fertilization and gamete specialization. Gamete transcriptomes showed a high level of complexity with a large portion of gender specific gene expression. Our results indicate that over 4,000 of expressed genes are differentially regulated between male and female, including sequences related to cell movement, carbohydrate and lipid metabolism, signaling, transport and RNA processing.

**Conclusions:**

This first comprehensive transcriptomic study of protist isogametes describes considerable adaptation to distinct sexual roles, suggesting that functional anisogamy precedes morphological differentiation. Several sex-biased genes and pathways with a putative role in reproduction were identified, providing the basis for more detailed investigations of the mechanisms underlying evolution of mating types and sexual dimorphism.

## Background

Sexual reproduction encompasses the fusion of two specialized haploid cells to form a zygote. Phylogenetic analyses suggest that sexual reproduction arose already in the common ancestor of all eukaryotes [1,2], implying the existence of selective forces that gave an advantage to sexuality. Despite the costs and major challenges presented to the reproductive cells, over 99.9% of eukaryotes engage in sex [3,4], which inspired generations of biologists to study this widespread phenomenon from physiological, molecular and evolutionary perspectives.

The existence of two gamete types and the tendency for gamete dimorphism remains an intriguing puzzle as anisogamy characterizes nearly all plants and animals. Oogamy (large eggs and small sperm) seemed to be the course of evolution from isogamy (equally-sized gametes) and arose independently in many groups of eukaryotes; however numerous species continue to reproduce with isomorphic germ cells [5,6]. Although substantial research addressed gene expression profiles in reproductive cells of flowering plants and animals [7-12], and studies of the mating locus in Volvocine algae shed light on the transition towards oogamy [13,14], not much is known about how the global patterns of sex-biased gene expression were shaped throughout the evolution of mating types and transition towards anisogamy. Such studies are important, because most of the evolutionary models accept the existence of two specialized mating types upon which the evolution of gamete size was superimposed [15]. Therefore, a detailed characterization of transcriptional adaptation in equally-sized gametes would bring a better understanding to the mechanisms underlying evolution of sexual dimorphism. In this respect, brown algae (Phaeophyceae) with their broad spectrum of gamete copulation forms are suitable subjects to test various hypotheses [16].

Brown algae are a large group of multicellular, photosynthetic organisms, which evolved 150–200 million years ago. Distant to land plants and animals, they developed complex multicellularity independently from other major clades [17,18]. This polymorphic group hosts seaweeds of a vast range of sizes, ecological niches and with an unmatched diversity of life cycles and fertilization strategies ranging from isogamy over anisogamy to oogamy [16,19]. Despite the evolutionary distance, brown algae share many common features with land plants, which first brought much attention to eggs and zygotes of brown macroalgae, due to their large size and abundance, as a material to study the regulation of early development in plants [20-22]. Other studies on reproduction have focused mainly on networks of signals that are associated with gamete attraction, recognition and fertilization success [23-26]. Although a large amount of research concerns brown algal biology, many aspects remain poorly explored, providing excellent opportunities for new discoveries.

In recent years, following the selection of *Ectocarpus* as a model for the brown algae, a considerable effort was invested in the development of genomic and genetic tools for this organism, among which was the assembly and analysis of the complete genome sequence [18,27]. *Ectocarpus* is a small filamentous alga, characterized by a haploid-diploid life cycle with isogamous sexual reproduction where flagellated gametes are still morphologically, but no longer physiologically, identical. Female gametes are distinguished by a short swimming period preceding settlement, flagella digestion and pheromone release [28]. Fertilization takes place immediately after recognition by gender specific sex-receptors present on the egg surface and the male anterior flagellum [29,30]. However, the dynamics and regulation of the mechanism driving male and female gamete differentiation and adaptation to fulfill their specific functions remain largely unexplored.

Here we describe transcripts specific to the gametes of both sexes. Using AB SOLiD 3 Next Generation Sequencing technology we generated whole RNA profiles of reproductive cells of *E. siliculosus* and determined the gender-specific regulation of the major metabolic pathways. The results present a first comparative gamete transcriptome analysis of any protist and provide an overview of the genes that contribute to the gametes’ cellular identity and function.

## Results and discussion

### Next Generation Sequencing and mapping of the *Ectocarpus* transcriptome

Sequencing of rRNA-depleted total RNA of *Ectocarpus* gametes yielded more than 36 million 50 bp reads for the male and 28 million 50 bp reads for the female sample. An overview of the results is shown in (Table 1). We were able to map on the genome 45 and 62% of male and female reads, respectively. Reads that had no match are likely to be PCR artifacts, were of low quality or have origins outside the reference genome. Since the *Ectocarpus* strain used in this study was different from the strain that was used for genome sequencing [31] it is also plausible that part of the unmatched sequences is derived from genomic variation between the two strains.

**Table 1:** **Transcriptome mapping results of *Ectocarpus siliculosus *** **male and female gametes**

	**Male gametes**	**Female gametes**
Total number of reads	36 751 768	28 591 842
Total number of mapped reads	16 580 350	17 697 894
High quality mappings (normodds > =0.7)	2 775 695	2 478 987
Mapped to known genes	899 347	1 386 445
Mapped outside of known genes	1 876 348	1 092 542

After the alignment quality assessment, 16.74% of all aligned reads for male and 14.01% of the reads for female were chosen as unique and high quality mappings to the nuclear genome. At this sequencing depth we found at least five non-clonal reads uniquely aligned to 8,029 and 7,777 of 16,239 annotated nuclear genes (male and female respectively). Summarized expression data is presented in Additional file 1: Table S1. Despite the high level of detection, more than 80% of the alignments fell outside the coding sequences. It is plausible that some of these come from intronic regions and are presumably descended from pre-mRNA present in the ribo-depleted total RNA sample or might represent intron retention events [18] since a large portion of these reads did not match annotated genes, indicating other transcriptionally active sites. These findings are in line with the whole genome tiling array, which identified 8,741 expressed regions longer than 200 bp outside of predicted genes as potential novel protein-coding regions or non-coding RNA genes [18].

**Figure 1:**
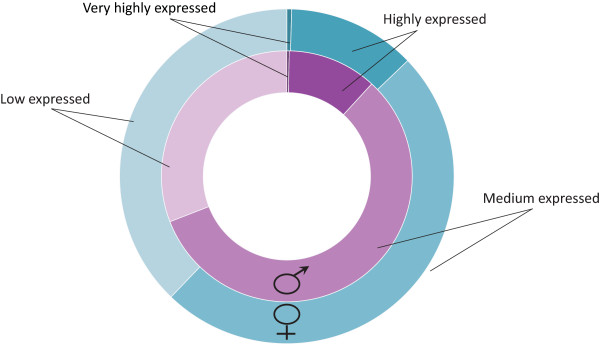
**Overview of gene classification into expression categories based on mapped reads number.** Genes were grouped into four categories: ‘Very highly expressed’ (read count > = 1000), ‘Highly expressed’ (1000 > read count > = 100), ‘Medium expressed’ (100 > read count > = 15) and ‘Low expressed’ (15 > read count > 5); male gametes (purple circle) and female gametes (blue circle).

The relative abundance of each gene in the mRNA pool was deduced by determining the Trimmed Mean of *M*-values (TMM) normalized number of reads mapped in the exon region and classified into four expression categories: Very highly expressed (read count > = 1000), Highly expressed (1000 > read count > = 100), Medium expressed (100 > read count > = 15) and Low expressed (15 > read count > 5). As shown in Figure 1 the sequencing data is enriched in medium and low expressed transcripts, confirming the sensitivity of our RNA-Seq approach to detect lowly expressed genes [32].

### Gametes have unique transcriptional profiles

**Figure 2:**
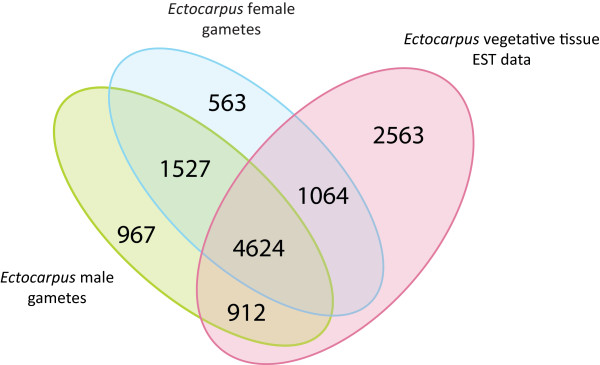
**Comparison of gene expression in gametes and vegetative tissue.** Venn diagram showing the overlapping genes that were found expressed in male gametes (8,029), female gametes (7,777) and *Ectocarpus* vegetative tissue EST represented genes (9,163) [33].

A Venn diagram (Figure 2) displays common gene expression between gametes and representative EST libraries [31] of vegetative gametophyte and sporophyte tissues (corresponding to 9,163 annotated genes). Approximately 70% of the EST sequences were shared by gametes and vegetative tissues. This is not surprising, since non-fertilized *Ectocarpus* gametes are capable of parthenogenesis and development into functional parthenosporophytes [28]. However, almost one-third of the gamete-expressed transcripts were found exclusively in the reproductive cells. Gene ontology (GO) analysis of this subset indicated that sequences related to signal transduction, RNA modification and localization and microtubule based movement were significantly enriched (p < 0.01). The high contribution of gamete-specific mRNAs within the whole transcriptome pool highlights the potential significance and regulatory specialization of this subset.

We also looked at the top 100 most expressed genes in gametes and manually grouped them into functional categories based on gene annotation information (Additional file 1: Table S2). The two largest functional clusters were composed of genes related to carbohydrate metabolism including cell wall biosynthesis (11 Female; 11 Male) and protein turnover (7 Female; 8 Male). However, the majority of the most abundant transcripts were of unknown function (60 Female; 55 Male).

### Functional classification of gamete-expressed genes

**Figure 3:**
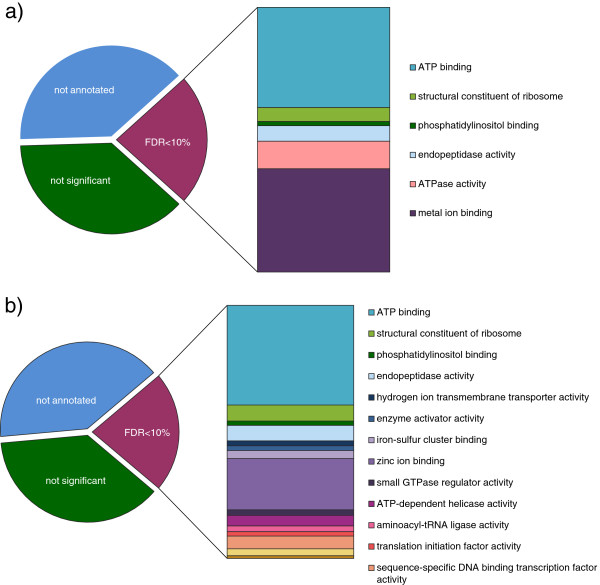
**Significantly enriched (FDR < 10%) Gene Ontology groups of gametes expressed genes according to molecular function. a)** Male gametes expressed genes; **b)** Female gametes expressed genes.

Around 62% of the gametes’ transcripts could be assigned with a Gene Ontology category using Blast2GO (E-value < 1e-05). Statistical analysis marked translation and gene expression, auxin biosynthesis, proteolysis, transport, localization and regulation of signal transduction significantly overrepresented in both gamete types (FDR < 10%). Additionally, we found that transcripts related to vesicle-mediated transport, lipid metabolism and iron/sulfur cluster assembly were significantly overrepresented in the female library, whereas sequences related to pigment biosynthesis were enriched in the male library. In the “cellular component” category, differences were observed in mitochondrion, nucleus, vesicle membrane and Golgi related components (overrepresented in female library) and chloroplast stroma (overrepresented in male library) (see Figure 3 for enrichment in Molecular Function and Additional file 1: Table S3 for full overview). In general, genes related to photosynthesis were underrepresented in the transcriptome of both gametes. Female gametes were also deficient in sequences related to DNA metabolic processes (e.g., protein-DNA complex assembly, nucleosome organization) as well as microtubule-based movement and male gametes had underrepresented genes in the cellulose binding group.

Based on significant similarity (E-value < 1e-05) we also assigned 2,418 and 2,243 Kegg orthology terms to gamete’s expressed proteins (male and female respectively) using the KOBAS server [34]. A significant proportion of the transcripts in females (FDR < 10%) were associated with ribosome, spliceosome and endocytosis. Ribosome was the only valid pathway overrepresented in male gametes with the given threshold (FDR < 10%).

### Analysis of differentially expressed genes

**Figure 4:**
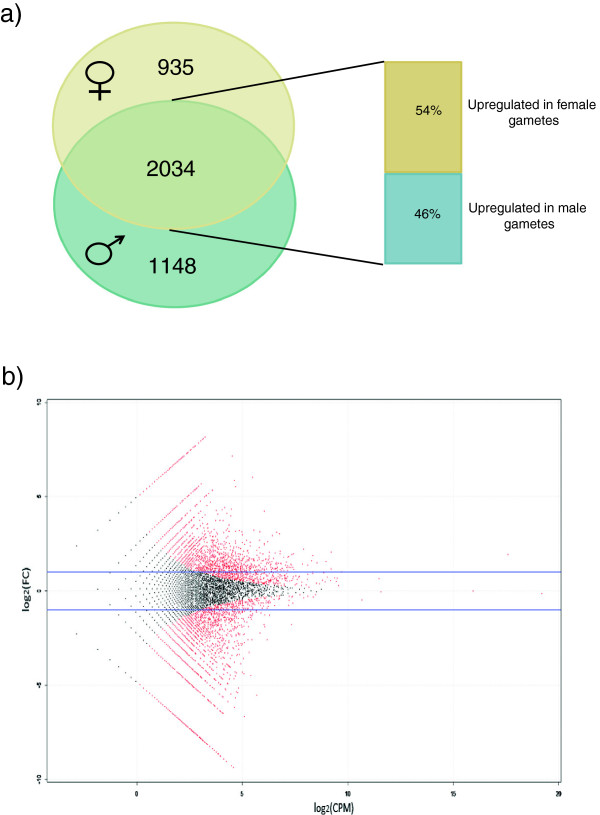
**Differential gene expression between male and female gametes of *Ectocarpus*. a)** Venn diagram showing the distribution of 4117 differentially expressed genes between gametes (Fold Change > = 2; FDR < 5%), with 935 and 1148 genes being found expressed only in female and male gametes respectively. Out of the 2034 genes expressed in both gamete types, 54% are upregulated in females. **b)** Scatter plot showing distribution of fold-change in expression in male versus female gametes (y-axis) against expression level (x-axis). Differentially expressed genes at significance level of FDR < 5% are colored red; blue line indicates 2-fold difference in expression.

**Figure 5:**
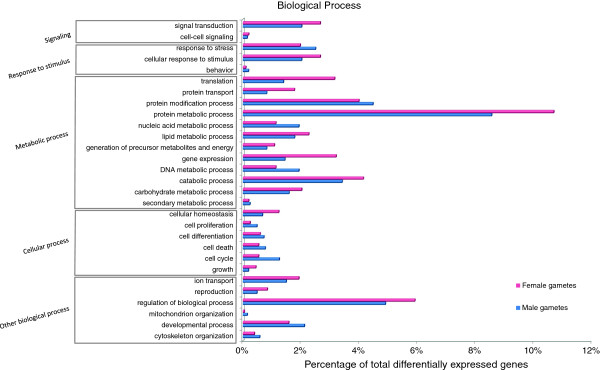
**Functional classification of differentially expressed genes according to biological process GO slim categories.** Genes were considered differentially regulated if the fold change was > =2 and FDR value was <5%. Values are expressed as percentage of genes in each differentially expressed group.

**Figure 6:**
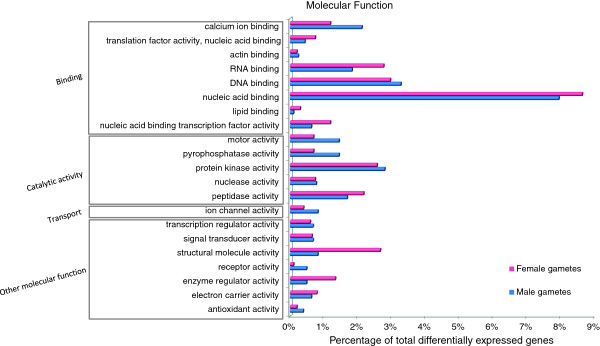
**- Functional classification of differentially expressed genes according to molecular function GO slim categories.** Genes were considered differentially regulated if the fold change was > =2 and FDR value was <5%. Values are expressed as percentage of genes in each differentially expressed group.

The preferential expression of genes belonging to a specific functional category became more evident when differentially expressed genes were considered. Using the edgeR package for R (FDR of 5% and a fold change > = 2) 4,117 genes were identified as differentially expressed between male and female gametes (Figure 4, Additional file 1: Table S4). Apparent enrichment could be seen in particular with the categories microtubule based movement, vesicle trafficking, ion dynamics, cell wall biosynthesis, transcription and translation regulation, and signaling related genes, which are described below (Figures 5 and 6, Additional file 1: Table S5; for details about involved genes see Additional file 1: Table S6).

### Microtubule based movement

The terms overrepresented in male gametes are assigned primarily to microtubule based movement (GO:0007018), which can be associated with the sperm active swimming behavior. Besides the canonical role in locomotion, *Ectocarpus* flagella are also important sensory organs involved in chemosensation and gamete recognition [25,30]. Several genes belonging to the intraflagellar transport (IFT) and motor protein families were overexpressed in male gametes. IFT proteins are macromolecular rafts responsible for the assembly and maintenance of the flagella [35] and the deposition of mastigonemes on the flagella surface after their assembly in the ER [36]. Growing evidence suggests IFT plays a more direct role in cilia-mediated signaling [37]. Among other genes linked to the gamete flagella, we found members of the Sexualy Induced Gene family – Sig1 and Sig2-like gene, with Sig1 among the highly overexpressed genes in male gametes. This family of proteins was first described during the onset of sexual reproduction in the diatom *Thalassiosira*[38] and later also in other Stramenopiles [39]. Sig proteins are located to the mastigonemes [40], but their function remains unresolved. However, striking diversification of Sig1 between closely related species of *Thalassiosira*[41] and some evidence of positive selection acting on this gene [42], may suggest a role in gamete recognition.

### Ion dynamics

Potassium channel activity (GO:0005267) and calcium-activated potassium channel activity (GO:0015269) were significantly overrepresented in male gametes. These might be related to the sperm chemotaxis in analogy to the gene network triggering motility response to stimuli in sea urchin sperm (see [43,44] for a review). It has been shown that cyclic nucleotide messengers and changes in K^+^ ion dynamics lead to hyperpolarisation of the cell membrane and activation of the Na^+^ and Ca^2+^ influx in sperm [45-50]. Recently, a transient increase in Ca^2+^ in the flagellum was directly visualized during chemotactic orientation in ascidian sperm using a fast Ca^2+^ imaging system [51]. Previous studies on *Ectocarpus* pheromone response confirm participation of free Ca^2+^ in sperm navigation, since concentrations below 10^-7^ M caused male gamete immobilization despite the presence of the attractant [52]. Highly upregulated male genes, with a homology to the *Strongylocentrotus* sperm pathway, included three cyclic nucleotide binding K^+^ channels with similarity to the TetraKCNG channel (BLAST E value >1e-20), a Na+/H + exchanger, an adenylate cyclase, a sperm hyperpolarization-activated and cyclic nucleotide-gated channel (BLAST E value >1e-15) and a similar voltage gated-calcium channel (BLAST E value >1e-33).

### Cell wall/polysaccharide biosynthesis

Members of different carbohydrate biosynthesis pathways were predominantly upregulated in female gametes, which relates probably to primary cell wall biogenesis minutes after fertilization [23]. All enzymes involved in alginate synthesis [53] and three *Ectocarpus* cellulose synthases (CESAs) are highly overexpressed in female gametes, which is apparent with the brown algal cell walls being composed of alginate with a minor fraction of cellulose [54]. Apart from alginate and cellulose, sulfated fucans and phenolic biomolecules (phlorotannins) are secreted into the expanding cell wall [55]. We can assume that these compounds are also synthesized in gametes, since all fucosyltransferases (except from family GT24) and sulfotransferases (STs) (except Clade B) [53] were transcribed with some gender specificity. It is worth noting that one sulfotransferase (Esi0032_0064), related to metazoan STs involved in the biosynthesis of glycosphingolipids, was characterized by a much higher expression comparing to other STs in both gametes. Associated with lipid rafts, glycosphingolipids may act as intermediates in signaling the flow from outside to inside the cell [56]. Sulfated fucans and galactans are also reported to be involved in sea urchin fertilization. They act as inducers of the sperm acrosome reaction [57,58] by binding to the sperm receptor REJ (Receptor for Egg Jelly), a homolog of the human polycystin protein [59]. Interestingly, five of the expressed sulfotransferases were specific only to female gametes and five polycystin/REJ-like proteins (IPR002859) were expressed exclusively in males.

### Vesicle trafficking

Gametes and spores of *Ectocarpus* can be characterized by the presence of several active Golgi bodies [60,61]. Similar observations were made with early electron scanning photographs of *Fucus* unfertilized eggs, which show a characteristic, rough surface due to protrusion of the cytoplasmic vesicles beneath the plasma membrane [23]. These findings are reflected in the upregulated genes of female gametes, since clathrin coat proteins (GO:0030118) constituting vesicles travelling from the Golgi apparatus to the plasma membrane [62] are highly abundant. The same is observed with the retromer complexes (GO:0030904), which assemble on early endosomes and are involved in transport back to the Golgi apparatus. Additionally, the Rab protein signal transduction pathway (GO:0032483), including the Arf family, which are coat-recruitment GTPases (for a recent review, see [63,64]) and dynamins, which are necessary for pinching the vesicles [62], are upregulated in female gametes.

The Golgi complex is the major site for polysaccharide synthesis including alginates, sulfated fucans and phlorotannins of the algal cell wall, which are transported in vesicles to the plasma membrane [55,65]. Thus, the upregulated pathways of cellulose biosynthesis and vesicular transport can support primary cell wall biogenesis. It is also possible that this secretory activity may be important for biosynthesis of the adhesive substance required for gamete attachment to a substrate [66].

### Translation and transcription regulation

Gene expression in gametes is developmentally regulated and stage specific, and thus requires a precise and well-coordinated program of transcription regulation. Gametes express transcription factors across most of the described TFs families in *Ectocarpus*[67]. In particular, we find members of Heat shock (HS) factors, fungal TR and CCAAT-binding overrepresented in females and most MYB genes overexpressed in males. Interestingly several MYB factors were also relatively high expressed in *Arabidopsis* sperm cells [9]. Among the highest expressed TFs we find two MYB factors (Esi0038_0132, Esi0212_0014), Zinc-finger C2H2-type factor (Esi0226_0040) and two fungal TRFs (Esi0008_0230, Esi0348_0008). Esi0212_0014 shares 53% identity (8e-37) with *Arabidopsis* MYB98 which controls the formation of specific features within the synergid cell during female gametophyte development [68,69].

Another interesting transcription factors family described by [18] is the NIN-like proteins, coded by nine genes in *Ectocarpus*. NIN-proteins are required for symbiosis between legumes and nitrate assimilating bacteria, and a subfamily of NIN, the minus dominance proteins (MID), are expressed during gametogenesis in volvocine algae and determine the minus mating type [70]. It was suggested, that the NIN-like family might have a role in life cycle or in mating type determination in *Ectocarpus* gametes [18]. Indeed eight members of this family are found in our data, with two of them being specific for female gametes. It is also worth noting, that the most highly expressed NIN factor in both gametes (Esi0013_0140) was significantly downregulated in the *immediate upright* mutant, which is defective in sporophyte development [18].

Protein metabolic processes, in particular biosynthetic pathways (ribosome and translation related) are significantly enriched (p < 0.01) in female differentially expressed genes. Additionally, *Ectocarpus* was shown to have a micro RNA post-transcriptional regulation system, where most of the mature miRNA bare a signature preferred by the plant Argonaute-1 protein (AGO1) [18]. The genome contains one AGO1 protein which is expressed at moderate level in both types of gametes. The Argonaute-miRNA are known to silence transcription, trigger target destruction, or inhibit translation, and growing evidence supports their role in germline development [11,71]. Moreover, several potential target sequences including members of the ROCO family GTPases and other proteins containing leucine-rich repeat (LRR) domains [18] are expressed in gametes. Since gametes are vulnerable targets for pathogen attack [72,73], these proteins might be involved in algal immune response to disease [74].

### Signaling pathways

Gamete transcripts are enriched in Ras GTPase superfamily genes (Ran, Ras, Rab, Rho and Arf). These signaling molecules are binary switches in crucial cellular processes including growth, differentiation and survival [75]. Rab and Arf are particularly important in membrane trafficking and are enriched in female gametes (see Vesicular transport). The Rho family is involved in signaling networks that regulate actin, cell cycle progression, and gene expression. Noteworthy RAC, a Rho family GTPase, and its positive effector RhoGEF are upregulated in females, whereas RhoGAP, a negative regulator, is highly expressed in male gametes. Rho genes, RhoGDI (guanine nucleotide dissociation inhibitor of Rho) and a RhoGAP were also expressed in *Arabidopsis* sperm, where no RhoGEFs were found [9]. The precise function of Rho signaling in sperm and egg remains to be revealed, but substantial work has been done by Kumakiri *et al.*[76], showing a role in initial sperm-egg fusion in mouse. In the study, *Clostridium difficile* toxin B inhibited sperm incorporation probably by disturbing actin filament reorganization regulated by Rho GTPases. Rac1 seemed to be strongly expressed in mouse eggs and located in the cortical ooplasm. The process of sperm–egg fusion in mouse would be initiated immediately after sperm binding by membrane receptors that in turn would activate Rho proteins by RhoGEFs. Membrane receptors activating RhoGEFs include G protein-coupled receptors, such as the lysophosphatidic acid (LPA) receptor, the growth factor receptors with a tyrosine kinase domain, such as EGF receptors, and surface proteins such as integrins [77]. Members of all these receptor families could be identified in *Ectocarpus* gametes, but the specific recognition protein involved remains unknown.

Other regulators of Rho GTPase activity (GO:0032319) are expressed in male gametes, including two Target Of Rapamycin (TOR) kinases (TOR1 and TOR2). TOR is a nutrient-sensitive, central controller of cell growth and aging, which was linked to the actin cytoskeleton via a signaling pathway containing a Rho GTPase [78]. Raptor and FKBP12, TOR associated proteins, are also expressed, but no RAG GTPases that promote intracellular localization of TOR were present. In yeast TOR kinases (TOR1 and TOR2) were shown to act in two different pathways (for a recent review see [79]). One pathway involved in cell growth in response to nutrient availability is shared between TOR1 and TOR2; however, TOR2 has additional, unique functions in sphingolipid synthesis, endocytosis and polarized organization of the actin cytoskeleton. *Ectocarpus* has two TOR kinases, both of which are expressed in gametes. Fold change expression analysis shows TOR2 to be upregulated in male gametes (FC = 8) compared to TOR1 (FC = 1.2), which could point to the importance of the second branch of TOR2-signaling in males, especially with high expression of glycosphingolipid-related ST (see Polysaccharide biosynthesis).

Ras GTPases influence transcription of genes involved in cell growth and division by activating protein kinases, such as the mitogen-activated protein (MAP) kinase. Several members of the family were detected in *Arabidopsis* sperm cells and some are sperm specific, implicating the existence of unique signaling pathways [9]. Out of five MAPKs expressed in gametes, two were upregulated in males. We found also one MAPK related serine/threonine protein kinase specific to male gametes, with homology to the LF4 gene (MAPK) localized in *Chlamydomonas* flagella [80]. This protein is involved in a signal transduction cascade controlling flagellar length.

Another effector activated by Ras is phosphoinositide-3-kinase (PI3K). There are two putative PI3Ks in *Ectocarpus*, one highly expressed in both types of gametes and one upregulated in males. The product of PI3K, phosphatidylinositol 3-phosphate, plays an important role in regulating membrane trafficking. Additionally, we identified two enzymes necessary for phosphoinositide-mediated signaling which were enriched in male gametes, phosphatidylinositol 4-kinase and 1-phosphatidylinositol-4-phosphate 5-kinase, involved in synthesis of phosphatidylinositol 4,5-bisphosphate (PIP2). PIP2 is a minor constituent of the plasma membrane, where it functions as an intermediate in a number of signaling pathways, including G protein-coupled receptor (GPCR) signaling. The sperm-induced breakdown of the PIP2 via activation of phospholipase C is considered to be the major reaction of fertilization [81,82]. The importance of the PIP2 secondary messenger system in sexual reproduction was shown in echinoderms eggs, where it regulates Ca^2+^ release at fertilization and controls the slow polyspermy block [83-85]. PIP2 was also abundant in the plasma membrane and the flagellar membrane of *Chlamydomonas eugametos* gametes, indicating involvement of phosphatidylinositol-calcium signaling system during mating, which could be activated by binding of cell-cell recognition receptors [86]. Additionally, alternative Ca^2+^ gates like ryanodine receptors may be involved following the propagation of a calcium wave [83,87]. One member of inositol triphosphate/ryanodine-type receptors is represented in *Ectocarpus* and found highly expressed in both types of gametes. Existence of inositol 1,4,5-trisphosphate-induced Ca^2+^ waves has been reported in *Fucus* embryos, emphasizing the importance of calcium signaling in response to a physiological stimulus [88,89]. One of the current hypotheses about sperm induced oocyte activation assumes stimulation of a membrane receptor that involves G protein signaling [90]. G-protein coupled receptors (GPCRs) are transmembrane proteins that utilize interactions with heterotrimeric G proteins (Gα, Gβ and Gγ) for downstream signaling and the pathway depends on the isoform of the α-subunit to which the receptor is coupled [91,92]. Six paralogs of the Gα subunits (GPA) are found in the *Ectocarpus* genome and are all expressed in gametes. GPA4 and GPA6 are among the highly transcribed genes whereas the GPA3 and GPA4 are overexpressed in male gametes. Moreover, three putative GPCR receptors are specific to male gametes and three partial GPCRs are upregulated in females. Substantial evidence supports a role of GPCRs in egg-sperm interactions during fertilization. For example, a G-protein coupled receptor located on the spermatozoa plasma membrane activates a signaling pathway responsible for the zona pellucida induced acrosomal exocytosis [93]. A G-protein coupled cAMP transduction pathway is also involved in chemotaxis in human sperm [94] and Gα proteins together with adenylyl cyclase were shown to be enriched in sea urchin sperm [95].

The GPCR receptor family is a host to many pheromone receptors [96]. It was shown that *Ectocarpus* sperm chemotaxis is stimulated in a similar manner as for pheromones by trifluoperazine (TFP) [52], which is an antagonist of dopamine/adrenergic G-protein coupled receptors. Thus, it might be possible that TFP activates the ectocarpene receptor which could belong to GPCR family.

Male and female gametes express genes related to Hedgehog and Notch signaling pathways involved in animal development (for a review see [97,98]). Although these pathways do not exist in a canonical form in non-metazoans, it has been shown that components such as the γ-secretase complex, Notchless and Hog/Hint domain proteins are of ancient origin [99,100] and new receptors seem to evolve by shuffling of pre-existing domains. The presence of Notch receptor building blocks is revealed in the *Ectocarpus* genome, however no homologues of the Notch receptor or its ligands *sensu stricto* have been found. A KEGG orthology analysis of gamete transcripts identified Deltex, a Notchless homolog, Presenilin and Nicastrin from the γ-secretase complex, two histone deacetylase co-repressors and three co-activators of the DNA binding protein with one highly expressed putative histone acetyltransferase. Additionally, 16 genes with a Notch domain (IPR000800) including Esi0061_0098, described by Le Bail *et al*. [101] as downregulated in the *Ectocarpus* developmental mutant – *etoile,* were present. Regarding the Hedgehog pathway, nine genes with similarity to Patched receptor (Ptc) of Hedgehog (containing both Patched (IPR003392) and SSD (IPR00731) domains) were abundant in gametes and one gene with Hint (Hedgehog/Intein N-terminal domain (SMART00306)) was low expressed only in females. Ptc and hint-domain proteins as well as Nicastrin and Notch-domain containing proteins were present during sexual reproduction in pennate diatom *Seminavis robusta*[102], although their involvement in cell-cell interaction is unknown and awaits further research.

### Pheromone biosynthesis

Brown algal pheromones are C-11 hydrocarbon compounds derived from fatty acids [103]. Female gametes of *Ectocarpus* use arachidonic acid as a precursor of ectocarpene [104] and accumulate large reserves of phosphoglyceride PX, rich in arachidonic and eicosapentaenoic acid, in their plasma membrane [105]. The hypothesized synthesis pathway involves lipoxygenase to form a peroxidised lipid and hydroperoxide lyase (LOX) for cleavage at the peroxidized site to C-11 hydrocarbon and conjugated oxoacid as a by-product [103]. GO and KEGG analyses revealed few lipoxygenases upregulated in female gametes, but no homologues of hydroperoxide lyases (HPL). Two Allene Oxide Synthases (AOS) indicated as putative hydroperoxide-lyases in pheromone pathway by Cock *et al*. [18] were also not expressed. Nevertheless, it could be possible that *Ectocarpus* LOX exhibits a double activity, like PpLOX1 from the moss *Physcomitrella patens*. Lipooxygenase form *Physcomitrella* combines the function of hydroperoxidase liase and acts on 18–22 carbon chains substrates [106]. It is also significantly similar to Esi0424_0006 LOX from *Ectocarpus* (E = 6e-29 (43%)).

*Ectocarpus* eggs are significantly enriched in genes related to glutathione (GSH), namely glutathione synthases and glutathione S-transferase. Glutathione as a radical scavenger prevents damage of cellular components caused by reactive oxygen species (ROS), such as peroxides [107]. Thus glutathione synthesis might be female’s cytoprotective strategy against oxidative damage in the presence of peroxidized lipids during pheromone synthesis. Additionally, the presence of particularly arachidonic acid, which is accumulated as a pheromone precursor in *Ectocarpus*, induced glutathione synthesis in human fibroblasts [108].

### RT-PCR validation

Ten genes that were identified with a high level of significance were selected to confirm the RNA-Seq results via qRT-PCR. To find the best normalization genes for gamete libraries, we investigated the expression of housekeeping genes reported by Le Bail [109] for microarray experiments. After analysis with geNorm [110] dynein and ribosomal protein 26S showed the smallest relative stability M-value (M = 0.27) across male and female gamete samples and were selected for normalization. Real-time PCR results were in general consistent with the direction of relative expression changes obtained by RNA-Seq, with a Pearson coefficient R of log_2_ (Fold Change) equal to 0.53, indicating a positive correlation between qPCR and RNA-Seq data. However, differences in the exact fold change values were observed (Table 2). Furthermore, the most stable 'housekeeping genes' as identified by qPCR in a previous report [109] including ubiquitin conjugating enzyme (UBCE), alpha tubulin (TUA), actin related protein (ARP2.1) and translation elongation factor 1 alpha (EFIa), showed only statistically non-significant relative changes of <1.5-fold (log2-ratio <0.58) in expression.

**Table 2:** Validation of SOLiD based gene expression profiles by Real-Time PCR

		**Log**_**2 **_**(Average relative expression)**^**a**^		
**Gene name**	**Gene function**	**RT-PCR**	**SOLiD**	**Validation (yes/no)**
Esi0102_0070	Arf1, ARF family GTPase	−1.93	−2.05	Y
Esi0067_0029	long chain acyl-coA synthetase	−4.19	0.49	N
Esi0069_0059	Mannuronan C-5-epimerase	−2.98	0.06	N
Esi0101_0018	Tubular mastigoneme-related protein	6.16	4.72	Y
Esi0104_0023	GPCR-like protein	4.72	2.22	Y
Esi0130_0068	PKD/REJ-like protein	0.62	2.22	Y
Esi0418_0017	MORN motif precursor	4.95	32.19	Y
Esi0123_0020	hypothetical protein	−7.15	−31.61	Y
Esi0161_0002	Metal ion transporter-like protein	3.50	−32.10	N
Esi0098_0063	hypothetical protein	−4.92	−31.61	Y

^a^Relative expression is calculated as a ratio of expression levels in male/female gametes to indicate genes up- or downregulated in male gametes. A gene is considered differentially expressed if its relative expression is twofold or greater.

## Conclusions

Here we provide the first to our knowledge, comparative analysis of protist gametes’ transcriptomes. One of the key findings of this study is that *Ectocarpus* gametes equal the intricate transcriptomes of oogamous species [9,11,111-113]. Most of the transcribed genes may not have an evident role before fertilization, nor are they necessarily translated, but they may be crucial during post-fertilization development as in plant and animal systems [114-116]. A large set of the expressed genes is common to somatic tissues, which implies their core metabolic functions and presumably also a role in the parthenosporophyte development. However, 4,117 genes in the *Ectocarpus* gametes’ transcriptome are differentially regulated and one-third of the identified transcripts seem to be gamete specific, with primary functions in signal transduction and RNA processing. It is remarkable that within the morphologically identical isogametes, the transcriptome profile is substantially divergent, reflecting the early establishment of distinct sexual roles. Both males and females are able to regulate levels of mRNA engaged in many cellular processes. The female transcriptome is depleted in genes related to chromatin organization and enriched in genes with function in cell wall biogenesis, vesicular transport, lipid metabolism with pheromone synthesis, gene expression and signaling. In male gametes a significant part of the upregulated genes relates to microtubule based movement and ion flux as well as signal transduction. These results can be linked to previously described gamete characteristics in *Ectocarpus* and sister species, like active swimming in males with a tight relation to ion dynamics [52], vesicle protrusions in settled females [23] pheromone production [25] and DNA dispersion in egg nuclei [23,117]. In addition, our data confirm that transcripts related to cell wall biogenesis are deposited in female gametes before fertilization. We also revise the proposed pheromone pathway and imply the potential role of glutathione in maintaining the cell oxidative balance. The comparative RNA-Seq analysis presented here revealed a number of signaling pathways potentially involved in gamete recognition and fertilization. In particular, genes related to phosphatidylinositol signaling, GPCR receptors, REJ-like proteins and sulphonyltransferases were found, providing new insight into the mechanism of gamete coupling. Similar results, with female-biased genes related to carbohydrate metabolism and male-specific transcripts with role in signaling were obtained when reproductive tissues from an oogamous brown alga *Fucus vesiculosus* were investigated [118]. Furthermore, the identification of differentially expressed transcription factors (like MYB or NIN-proteins) brings potential for discovery of sex specific gene expression regulators.

Taken together, we demonstrated a highly functional specialization in morphologically identical isogametes of *Ectocarpus*. Further insights into activated genes and pathways regulating gamete differentiation will result not only in better understanding of these reproductive cells and their interactions during fertilization but may also link sex determination to the formation of functional male and female gametes and shed light on the forces shaping the evolution of different sexes.

## Methods

### Culture conditions and gamete harvesting

*Ectocarpus siliculosus* (Ectocarpales, Phaeophyceae) unialgal strain NZKU 1–3 male gametophyte (CCAP 1310/56) and NZKU 32-22-21 female gametophyte obtained from a meiospore of NZKU z32 (CCAP 1318/85) were cultivated at 12°C in natural sea water enriched with modified Provasoli ES [119] with 14 h light/10 h darkness cycles (30 μmol × m^-2^ × s^-1^ flux density). Both gametophytes descend from a single diploid sporophyte collected in Kaikoura, New Zealand representing ‘*Ectocarpus* lineage 4’ according to [120]. To induce gamete release fertile gametophytes were transferred to Petri dishes with residual water only and kept overnight at 4°C in the dark. Gamete release was induced by immersing cultures in PES in direct light at room temperature. Gametes were collected using a micropipette, transferred into 1.5 ml Eppendorf tubes and pelleted at 5,000 × g for 5 minutes. Gamete pellets were flash-frozen in liquid nitrogen and stored at -80°C before RNA extraction.

### RNA extraction and sequencing

Total RNA was isolated using an XS RNA extraction kit (Machery-Nagel) or RNeasy Plant Mini kit (Qiagen) according to manufacturer’s instructions. An additional DNase digestion step was performed in solution with RNase Free Turbo DNase (Ambion). The concentration and purity of all samples was measured with a Nano-Drop spectrophotometer (ND-1000, Thermo) and the sample integrity was checked on a 1% agarose gel. Approximately 20 μg of total RNA from each type of gamete was rRNA depleted and shredded prior to cDNA synthesis using the SOLiD™ Total RNA-Seq Kit. Male and female samples were barcoded and prepared cDNA libraries were pooled and sequenced with a SOLiD 3 System (Applied Biosystems) at Cofactor Genomics (Missouri, USA).

### Mapping reads to the reference genome

SOLiD sequence reads were trimmed from adaptors and filtered for full 50 bp length. Reads were mapped to the reference genome [121] using SHRiMP2 [122] with a threshold score for full Smith-Waterman alignment set to 60%. Raw sequence data were first aligned against the *Ectocarpus siliculosus* rDNA sequences to check for depletion efficiency (rRNA contamination was estimated approximately 0.55% for female and 0.12% for male library) and then to the *Ectocarpus* genome. With the observed base quality drop towards the read’s end and considering that the sequencing data were obtained from a different strain then the sequenced one, we used less stringent conditions for alignment scores and filtered reads based on mapping quality parameters. The statistical significance of top scoring hits was calculated using the Probcalc module of SHRiMP2 and only unique mappings with ‘normodds’ value > =0.7 were selected. The same filtering parameters were used to align raw data against the mitochondrial and chloroplast genome of *Ectocarpus*. Additionally Tophat software [123] was used to identify reads mapped in exon-exon splice junctions, allowing 1 mismatch and an intron length of maximum 5000 bp.

### Estimation of transcript abundance and differential gene expression analysis

We used HTSeq-count [124] to locate and count aligned reads within annotated genes, based on the available *Ectocarpus siliculosus* gene set at the OrcAE platform (http://bioinformatics.psb.ugent.be/orcae/overview/Ectsi). We also determined the number of reads mapped in exon, intron, 3’ UTR and 5’ UTR regions. Only exon mapped reads were considered in further analysis. Read processing involved filtering based on the number of reads per CDS, the covered length, and those with less than 5 reads mapped or covering less than 51 bp were discarded. These data were compiled into the gene expression table that served as input into the edgeR package for R [125]. Library normalization was done using the trimmed mean of M-values method (TMM) [126] and Exact-Test was used to determine differentially expressed genes with P = <0.01 and FDR = <0.05.

### GO and KEGG enrichment analysis

To classify expressed genes, all sequences were annotated with KEGG orthology using KOBAS [34] and Gene Ontology (GO) categories using Blast2GO [127]. These automatic annotations were used to investigate overrepresented pathways and GOs by comparison of individual libraries to all annotated genes in *Ectocarpus*. Over-expressed KEGG pathways were identified using the KOBAS web-platform [34] and a hypergeometric test with Multiple Testing Correction of FDR [128]. Over-represented GO terms were identified with Blast2GO and Fisher's Exact Test with Multiple Testing Correction of FDR [128].

### Validation of RNA-Seq data by qRT-PCR

Quantitative real-time PCR was used to validate differential expression of ten selected genes (Table 2) and primers were designed using Primer3 software [129] with default settings (Table 3). cDNA synthesis was carried out on 1 μg of total RNA samples using oligo(dT)_12–18_ primer (Invitrogen) and GOScript reverse transcriptase (Promega) according to the manufacturer’s instructions. The qPCR reactions were performed in a 384-well thermocycler (LightCycler 480, Roche) with SYBR green chemistry (LightCycler 480 SYBR Green I Master mix, Roche) using listed conditions: 15 min at 95°C, followed by 40 cycles of 15 sec at 95°C, 20 sec at 50°C, and 30 sec at 72°C. Two biological replicates were run for both male and female cDNA samples and each sample was technically duplicated. Amplification specificity was measured with a melting curve by heating the sample from 65 to 97°C and the product size was checked on 1% agarose gel with GeneRuler™ 1 kb DNA Ladder (Fermentas). Absence of contaminating genomic DNA was checked with No-RT control PCR prior to cDNA synthesis. Normalization genes were selected using geNorm [110] and the relative gene expression values were calculated in qBASE v.1.3.5. [130].

**Table 3:** PCR primers used in this study for Real-Time PCR experiments

	**Oligonucleotides**		
**Gene name**	**Forward**	**Reverse**	**PCR product length [bp]**
Esi0102_0070	CTCAGCACTGCAGTCGTTAC	CGCGATCCAAGTGTACAAGG	166
Esi0067_0029	GCTGAAGTATCTCGACGGGA	TCTCATCGTACGGTCAACCC	220
Esi0069_0059	GAGATGCAACAACGTCGAGA	TCGAACGTGTTGTTGGTGAT	249
Esi0101_0018	AGATCAAGCTGGACAGGC	TGTGTATCGCAGTTCTCATT	253
Esi0104_0023	CCAACGCTCAGGTTCGCA	CCGTCCATGGCTCTCTCT	220
Esi0130_0068	ATCGGGGCCTTTCTCTCC	TGAAGGGAAGATCGCGATTC	147
Esi0418_0017	TTTGAGGGTGGCAAATAACC	CGTGTTTCTCTCCCTTCTCG	212
Esi0123_0020	CCTCCCTACGTCACCAAGAA	CACATCTTGTCGTCGTGCTT	239
Esi0161_0002	ACACAAGCCATTCCGATCAT	AGCGGGTACAACCATAAACG	182
Esi0098_0063	ATTGGCGTCGGGTTGTACT	TACCTTTCCGCATTGTGAGC	163
Esi0298_0008	ATGTCCGAAGACATGCAACA	TGGGTAACGTAGGACCCAAA	167
Esi0072_0068	GAACCACGGAAGGAACAAGA	GGAGGGCGTAGTTGTCGAAC	176

## Abbreviations

Bp: Base pair; PCR: Polymerase chain reaction; TMM: Trimmed mean of M values; GO: Gene ontology; KEGG: Kyoto Encyclopedia of Genes and Genomes; FDR: False discovery rate; IFT: Intraflagellar transport; CESA: Cellulose synthase; ST: Sulfotransferase; TF: Transcription factor; TFP: Trifluoperazine; GSH: Glutathione; ROS: Reactive oxygen species.

## Competing interest

The authors declare that they have no competing interests.

## Authors’ contributions

AL prepared the NGS libraries, performed the analyses of RNA sequencing data and drafted the manuscript. SD carried out the qRT-PCR experiments. ODC and EVD designed and coordinated the study and drafted the manuscript. All authors read and approved the final manuscript.

## Supplementary Material

Additional file 1: Additional file Table S1 List of genes expressed in *Ectocarpus* gametes. **Additional file Table S2.***Ectocarpus* gametes’ 100 most expressed genes. **Additional file Table S3.** Gene Ontology enrichment of gametes’ all expressed genes. **Additional file Table S4.** List of gametes’ differentially expressed genes. **Additional file Table S5.** Gene Ontology enrichment of gametes’ differentially expressed genes. **Additional file Table S6.** Genes associated with upregulated pathways described in Results and Discussion. Click here for file
